# Cat owners’ perceptions of multimodal environmental modification advice for obstructive feline idiopathic cystitis

**DOI:** 10.1177/1098612X251381483

**Published:** 2025-11-21

**Authors:** Kevin L Cosford, Sarah MA Caney

**Affiliations:** 1 Department of Small Animal Clinical Sciences, Western College of Veterinary Medicine, University of Saskatchewan, Saskatoon, SK, Canada; 2 Vet Professionals, Edinburgh, UK

**Keywords:** Multimodal environmental modification, MEMO, obstructive feline idiopathic cystitis, O-FIC, environmental enrichment, advice, client survey

## Abstract

**Objectives:**

The primary goal of this survey was to gain insight into cat owners’ perspectives with respect to multimodal environmental modification (MEMO) advice for obstructive feline idiopathic cystitis (O-FIC). The secondary goal was to describe the environmental management practices of cat owners.

**Methods:**

An online survey of clients whose cats were managed medically for presumed O-FIC was completed.

**Results:**

A total of 167 responses met the inclusion criteria. The proportions of cat owners receiving advice for MEMO varied with each aspect of environmental enrichment: diet (94%), water intake (86.2%), litter box (56.9%), private physical space (43.7%), social interaction (25.1%) and natural behavior (26.3%). For all these environmental enrichment categories, clients reported high compliance rates (88.9–97.6%), and veterinarians were the main source of advice. Cat owners indicated similar median satisfaction scores (with the thoroughness of advice), in the range of 77–82, for all aspects of environmental enrichment. Overall, respondents also reported encountering minimal challenges in terms of implementing MEMO and described their current management practices.

**Conclusions and relevance:**

Cat owners report that certain aspects of MEMO are emphasized for O-FIC, such as diet and water intake. Recommendations for litter box management, private physical space, social interaction and natural behavior are aspects of environmental enrichment that are not as commonly provided to cat owners. If given MEMO recommendations, cat owners readily complied. Veterinarians were the main source of MEMO advice, suggesting that they are the key to providing recommendations pertaining to all aspects of environmental enrichment.

## Introduction

Feline idiopathic cystitis (FIC), both acute and chronic forms, is a common problem encountered in feline medicine.^1,2^ FIC can also vary in its manifestation, being either non-obstructive or obstructive.^1^ In two retrospective studies from Norway^3^ and Germany,^4^ FIC was the most common diagnosis in cats with lower urinary signs, representing approximately 55% of cases compared with alternative differentials such as bacterial cystitis, urolithiasis and neoplasia.^3,4^ In North America, cats with lower urinary tract disease presenting to academic teaching hospitals represented anywhere in the range of 2–13% (mean 8%) of the feline caseload.^5^ Obstructive feline idiopathic cystitis (O-FIC) occurs in male cats, presumably as a result of urethral plugs consisting of struvite crystals, mucus/protein matrix, cellular debris and inflammatory cells.^1,6^ Feline urethral obstruction results in a life-threatening emergency necessitating appropriate treatment, which is associated not only with risks and welfare concerns for cats but also significant financial and caregiver burden for clients.

Stress, including that within the home environment, is believed to play a key role in the etiology of FIC.^1,2,7,8^ Many of these cats are young and living strictly indoors, which may not provide sufficient stimulation.^1,2,7,8^ Ultimately, deficiencies arise in psychological, physical and exercise needs.^7–9^ Prioritizing and optimizing feline health and animal welfare through environmental enrichment is essential to minimize stress-related problems such as FIC.^1,2,7,8^

To ensure that all the aspects of cats’ environments are being appropriately scrutinized, it has been proposed by Herron and Buffington^7^ to consider five basic ‘systems’: nutrition (diet and water intake), elimination (litter box), physical space, social interaction and engagement in natural behavior(s). Those authors suggest that successfully implementing multimodal environmental modification (MEMO) is possible by identifying environmental factors that could be improved upon, then working collaboratively with clients and coaching them to set realistic goals.^8^

The primary goal of this survey was to gain insight into client perceptions of advice they received with respect to MEMO after the initial episode of O-FIC. Using the aforementioned five basic systems, cat owners reported whether they received advice for environmental enrichment, the main source of that advice, their satisfaction with the thoroughness of the advice, whether they complied with the advice and any difficulties encountered when implementing the advice. We hypothesized that cat owners will have received and complied with advice for certain aspects of environmental management more commonly than others. Additional hypotheses were that cat owners would see veterinarians as the main source of advice for all aspects of MEMO and that they would perceive few challenges in implementing the recommendations. A secondary goal was to describe the environmental enrichment practices that cat owners were providing at the time of completing the survey.

## Materials and methods

A 65-question internet survey was conducted for cat owners with experience in urethral obstruction of a male cat. Links and QR codes to the survey were made available through veterinary medical associations in Canada, social media (a variety of Facebook groups [professional veterinary groups, feline foundations and client support groups]) and institutional websites (University of Saskatchewan, Vet Professionals). The platform was Survey Monkey, and responses were collected over 3 months (3 January to 3 April 2024). Survey questions consisted primarily of multiple-choice – both single (best fit) and multiple (all that apply) – answer formats. A slider scale between 0 and 100 (with hidden numerical input) was utilized to score cat owners’ satisfaction with the advice provided to them. Numerical input ‘0’ referred to the lowest satisfaction (least amount of information and guidance provided), whereas the numerical input ‘100’ referred to the greatest satisfaction (most amount of information and guidance provided).

The survey was designed in two parts. The first section evaluated the advice that cat owners received regarding MEMO. Survey questions focused on whether caregivers received and complied with advice, as well as the main source of the recommendation, and satisfaction with the thoroughness of the information provided. The second section evaluated cat owners’ current environmental management practices within the home. For both sections of the survey, all five basic aspects of environmental enrichment in the home as described by Herron and Buffington^7,8^ were emphasized, including nutrition (diet and water intake), elimination (litter box management), private physical space, social interaction and engagement in natural behavior(s) (see Table S1 in the supplementary material).

Participation was voluntary and anonymous. Withdrawal from the survey could occur at any time before data analysis. Participants granted permission for their data to be used for research, publication and teaching. Survey results were then downloaded into a Microsoft Excel spreadsheet (version 2501 Build 16.0.18429.20132) for data processing, descriptive statistical analysis and generation of figures. Duplicate and incomplete surveys were excluded. Cats undergoing perineal urethrostomy and/or cystotomy were also excluded. Although most questions were analyzed and included in this manuscript, those lacking sufficient responses were not evaluated further.

This survey was approved by the University of Saskatchewan Behavioral Research Ethics Board (Beh-REB #3534) and can be found in the supplementary material.

## Results

A total of 250 responses were collected. Only two IP addresses were duplicated, so the entry with the most complete response for each of these two cats was retained. A total of 36 submissions with incomplete responses were excluded. In total, 45 responses were excluded because a surgical procedure had been performed: 26 perineal urethrostomy (PU) cases, 10 cystotomy cases, and nine cases that had both PU and cystotomy procedures. A total of 17 responses to the treatment question indicated either ‘I don’t know/remember/unsure’ (four responses) or ‘Other’ (13 responses). It was concluded that these cases had undergone medical treatment – passage of a urinary catheter and no surgical intervention – based on comments provided to other questions. These 17 responses were retained for analysis. In the end, 167 total responses were retained for analysis.

Most respondents (120/167, 71.9%) were from Canada. Other countries from which multiple responses were obtained include the USA (25/167, 15.0%), the UK (11/167, 6.6%) and Australia (6/167, 3.6%). A single response (0.6%) was obtained from each of the following countries: Argentina, Guatemala, France, Peru and Portugal.

### Cat population

A summary of the descriptive data for the 167 cats medically managed for O-FIC is provided in Table 1. The duration of time during which 167 cat owners had been incorporating MEMO varied considerably: less than 2 months (n = 45, 26.9%), 2–6 months (n = 21, 12.6%), 7–11 months (n = 7, 4.2%), 1 year (n = 19, 11.4%), 2 years (n = 26, 15.6%), 3 years (n = 16, 9.6%), 4 years (n = 8, 4.8%), more than 5 years (n = 20, 12.0%) or unsure (n = 5, 3.0%).

**Table 1:** Descriptive data of 167 cats medically treated for obstructive feline idiopathic cystitis

Characteristics	Values
Age	
Kitten (0–6 months)	1 (0.6)
Junior (7 months–2 years)	29 (17.4)
Prime (3–6 years)	75 (44.9)
Mature (7–10 years)	34 (20.4)
Senior (11–14 years)	20 (12.0)
Super senior (⩾15 years)	8 (4.8)
Neuter status	
Neutered	161 (96.4)
Intact	6 (3.6)
Breed	
Domestic shorthair	96 (57.5)
Domestic mediumhair	18 (10.8)
Domestic longhair	16 (9.6)
Persian	4 (2.4)
Himalayan	3 (1.8)
Maine Coon	3 (1.8)
Mixed breed	3 (1.8)
Exotic Shorthair	2 (1.2)
Siamese	2 (1.2)
Bengal	1 (0.6)
British Shorthair	1 (0.6)
Munchkin	1 (0.6)
Norwegian Forest Cat	1 (0.6)
Ragdoll	1 (0.6)
Russian Blue	1 (0.6)
Scottish Fold	1 (0.6)
Siberian	1 (0.6)
Singapura	1 (0.6)
Snowshoe	1 (0.6)
Sphynx	1 (0.6)
Unsure	9 (5.4)

Data are n (%). All 167 respondents answered these questions

### Part 1: Cat owner perceptions of multimodal environmental advice

#### Advice received

**Figure 1:**
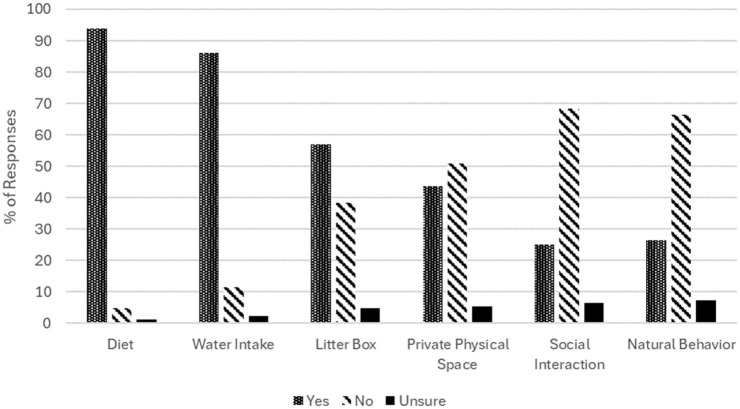
Percentage of cat owners given advice relating to six aspects of multimodal environmental modification. Percentage (%) = number of respondents who indicated that they were given advice/total number of respondents (denominator = 167 respondents)

Most of the 167 respondents received advice about diet (157, 94%), water intake (144, 86.2%) and litter box management (95, 56.9%) (Figure 1). Notably fewer respondents received advice regarding private individual space (73/167, 43.7%), social interaction (42/167, 25.1%) and natural behavior (44/167, 26.3%) (Figure 1).

#### Satisfaction with advice

**Figure 2:**
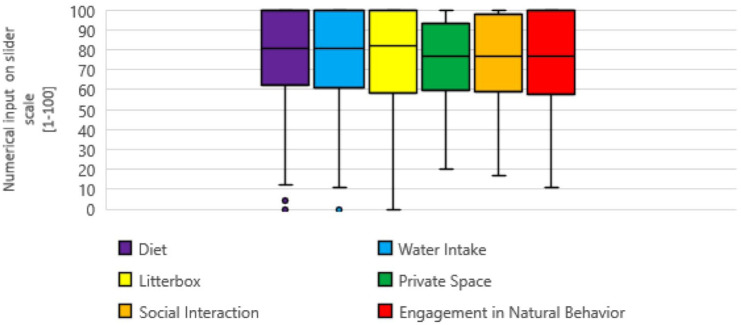
Cat owner satisfaction scores, on a slider scale of 0–100, for advice given related to multimodal environmental modification. Numerical input ‘0’ referred to the least satisfaction possible (least amount of information and guidance possible) whereas the numerical input ‘100’ referred to the most satisfaction possible (most amount of information and guidance possible). Box and whisker plots: median (central line inside box), box (first and third quartiles), whiskers (minimum value to first quartile on bottom and maximum value of the fourth quartile on top) and outliers (circles representing individual data points)

Cat owners provided high median satisfaction scores for the advice pertaining to all six aspects of MEMO: diet (n = 152, median 81), water intake (n = 139, median 81), litter box (n = 91, median 82), private physical space (n = 69, median 77), social interaction (n = 41, median 77) and natural behavior (n = 42, median of 77) (Figure 2).

#### Compliance with advice

**Figure 3:**
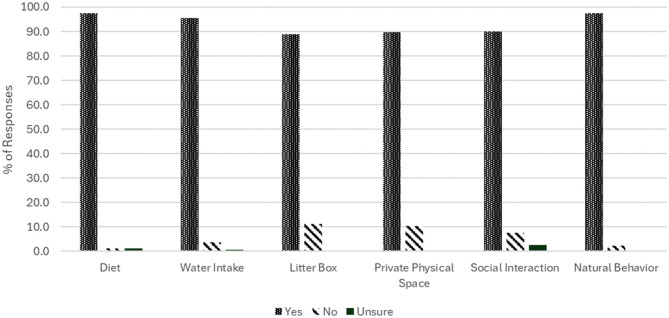
Percentage of cat owners complying with advice relating to six aspects of multimodal environmental modification. Percentage (%) = number of respondents indicating that they complied with advice/total number of respondents who received that advice

Most cat owners complied with advice given for all six aspects of MEMO: diet (147/151, 97%), water intake (132/138, 95.7%), litter box (80/90, 88.9%), private physical space (61/68, 89.7%), social interaction (36/40, 90.0%) and natural behavior (40/41, 97.6%) (Figure 3).

There were a small number of responses indicating reasons for not complying with advice: diet (n = 2), water intake (n = 5), litter box (n = 10), private physical space (n = 7), social interaction (n = 3) and natural behavior (n = 1). A summary of these reasons is in Table S1 in the supplementary material.

#### Main source of advice

The main source of advice for all six aspects of MEMO was the veterinarian: diet (111/155, 72%), water intake (81/142, 57.0%), litter box (60/93, 64.5%), private physical space (43/71, 60.6%), social interaction 21/41, 51.2%) and natural behavior (22/42, 52.4%) (Table 2). Other sources of advice are summarized in Table 2.

**Table 2:** Main source of information for multimodal environmental modification

	Diet	Water intake	Litter box	Private space	Social interaction	Natural behavior
Veterinarian	71.6	57.0	64.5	60.6	51.2	52.4
Veterinary nurse	8.4	9.9	14.0	9.9	7.3	9.5
Internet search	6.5	11.3	9.7	14.1	24.4	26.2
Online groups	6.5	7.0	4.3	5.6	7.3	4.8
Literature (paper)	1.3	3.5	2.2	4.2	7.3	2.4
Pet store staff	2.6	2.1	2.2	0	0	0
Word of mouth	1.3	1.4	1.1	1.4	2.4	2.4
Unsure	0	1.4	1.1	0	0	0
None of the above	1.9	6.3	1.1	4.2	0	2.4

Values represent percentages of cat owners indicating a primary or main source of advice

#### Challenges associated with advice

Most respondents indicated that they did not experience any difficulty implementing the modifications: diet (71/141, 50.4%), water intake (83/109, 76.1%), litter box (64/73, 87.7%), private physical space (45/56, 80.4%), social interaction (21/31, 67.7%) and natural behavior (28/37, 75.7%) (Table 3). Challenges associated with all six aspects of MEMO are summarized in Table 3. The biggest challenges to making implementations related to diet were expense (29.8%) and impracticality in multi-cat households (11.3%).

**Table 3:** Percentages (%) of cat owners reporting difficulties applying to all six aspects of multimodal environmental modification

	Diet	Water intake	Litter box	Private space	Social interaction	Natural behavior
No challenges	50.4	76.1	87.7	80.4	67.7	75.7
Changes not accepted						
At first	18.4	16.5	4.1	5.4	29.0	18.9
At all	1.4	2.8	2.7	1.8	3.2	0
Expensive	29.8	1	1.4	3.6	0	5.4
Time commitment (too great)	2.1	0	0	3.6	6.5	0
Multiple cats (impractical)	11.3	1	1.4	1.8	6.5	2.7
Overwhelming options	2.8	0	0	0	3.2	2.7
Inadequate information	0	1	0	1.8	3.2	2.7
Unsure	0	2.8	2.7	1.8	3.2	2.7

Percentage (%) = number of respondents indicating a specific challenge applied to them/number of respondents who complied with advice for each aspect of multimodal environmental modification and subsequently answered this question

Challenges associated with specific aspects of MEMO are summarized below:

- Diet: of 141 cats, 19 (13.5%) required mixing of different foods to coax the cat to eat, five (3.5%) had a concurrent health problem making dietary changes difficult, 10 (7.1%) vomited during the diet change and one (0.7%) had diarrhea during the diet change.
- Litter box: of 72 cat owners, two (2.7%) had limited space for additional litter boxes.
- Private physical space: of 56 cat owners, two (3.6%) stated that they had limited physical space in the home to implement these modifications and two (3.6%) were limited by the fact they lived in a rental property.

### Part 2: Cat owner-reported multimodal environmental modification practices

#### Diet management

Detailed cat owner-reported dietary management practices are summarized in Table 4. Most respondents fed a single diet type (115/132, 87.1%), with veterinary prescription diets accounting for 62.9% (n = 83). With respect to dry vs wet food, most respondents (88/127, 69.3%) fed a mixture of both dry and wet food. In terms of urinary vs non-urinary diets, most respondents fed a diet formulated for the urinary system (86/126, 68.3%).

**Table 4:** Cat owner-reported dietary management practices

Diet types (n = 132)	
Single	115 (87.1)
OTC commercial diet	27 (20.5)
Veterinary prescription diet	83 (62.9)
Raw diet	5 (3.8)
Cooked homemade diet	0 (0)
Combination of different types	17 (12.9)
OTC commercial/veterinary prescription	8 (6.1)
OTC commercial/raw	2 (1.5)
Veterinary prescription/raw	2 (1.5)
OTC commercial/raw/cooked homemade	1 (0.8)
OTC commercial/cooked homemade	2 (1.5)
Veterinary prescription/cooked homemade	1 (0.8)
OTC/veterinary prescription/cooked homemade	1 (0.8)
Moisture content of diets (n = 127)	
Dry (kibble)	24 (18.9)
Wet (tin cat, semi-moist, foil tray, pouch)	15 (11.8)
Mixture of both	88 (69.3)
Urinary vs non-urinary diets (n = 126)	
Urinary formulation	86 (68.3)
Non-urinary formulation	15 (11.9)
Mixture of both	23 (18.3)
Unsure	2 (1.6)

Data are n (%). Values represent the percentage of cat owners indicating their management practices for each item

OTC = over the counter

#### Water intake management

Detailed cat owner-reported water intake management practices are summarized in Table 5. The use of water bowls as the only water source was most common, reported by 70/147 (47.6%) respondents. With respect to the number of water bowls relative to the number of cats in the home, 125 respondents reported the following: more bowls in 47 (37.6%) households, the same number in 53 (42.4%) and fewer in 25 (20.0%). Multiple locations for water bowls were reported by 85/125 (68.0%) respondents, while a single location was reported by 40/125 (32.0%) respondents.

**Table 5:** Cat owner-reported water intake management practices

Water source(s) (n = 147)	
Bowl(s)	70 (47.6)
Fountain(s)	22 (15.0)
Both	55 (37.4)
Water bowl(s)	
Water bowls relative to the number of cats in the home (n = 125)	
More	47 (37.6)
Same number	53 (42.4)
Fewer	25 (20.0)
Unsure	0 (0)
Number of locations for water bowls in the home (n = 125)	
Multiple	85 (68.0)
Single	40 (32.0)
Water bowl filled to the top (brim) or partially filled (n = 125)	
Filled	86 (68.8)
Partially filled	35 (28.0)
Unsure	4 (3.2)
Water bowl (⩾12 cm brim) to avoid contacting whiskers (n = 125)	
Yes	88 (70.4)
No	27 (21.6)
Unsure	10 (8.0)
Material(s) from which the water bowl(s) are made (n = 125)	
Usage of water bowls from a single material	68 (54.4)
Glass	6 (4.8)
Ceramic	28 (22.4)
SS	24 (19.2)
Plastic	9 (7.2)
Wood*	1 (0.8)
Usage of a combination of water bowls each from different materials	57 (45.6)
Ceramic/SS	15 (12.0)
SS/plastic	8 (6.4)
Glass/ceramic	8 (6.4)
Glass/ceramic/SS	6 (4.8)
Ceramic/plastic	5 (4.0)
Glass/SS/plastic	6 (4.8)
Glass/SS	2 (1.6)
Glass/plastic	1 (0.8)
Ceramic/SS/plastic	3 (2.4)
Glass/ceramic/SS/plastic	3 (2.4)
Water fountain(s)	
Water fountains relative to the number of cats in the home (n = 145)	
More	3 (2.1)
Same number	14 (9.7)
Fewer	62 (42.8)
No fountain(s)	66 (45.5)
Number of location(s) for water fountains in the home (n = 142)	
Multiple	19 (13.4)
Single	60 (42.3)
No fountain(s)	63 (44.4)
Additional ways to encourage water intake	
Allow a tap to drip (bathroom or kitchen) (n = 147)	
Yes	30 (20.4)
No	117 (79.6)
Use a hydration supplement (Hydra Care [Purina] or Oralade [Macahl Animal Health]) (n = 147)	
Yes	10 (6.8)
No	137 (93.2)
Flavor the water (tuna juice, other) (n = 147)	
Yes	16 (10.9)
No	131 (89.1)

Data are n (%). Values represent the percentage of cat owners indicating their management practices for each item

* One respondent indicated that they did not use water bowls made from any of the possible materials listed (none of the above). In an open comment box, the respondent indicated that they used water bowls made from wood

SS = stainless steel

#### Litter box management

The number of litter boxes in the home exceeded the number of cats in 59/147 (39.8%) households, was equal in 68/147 (48.5%) households and was fewer in 18/147 (11.7%) households. Litter boxes were placed in multiple locations in 68/147 (46.3%) homes and in a single location in 77 (52.4%) homes. Of the 147 respondents, only two (1.4%) reported not using a litter box at all.

#### Private physical space management

Detailed cat owner-reported private physical space management practices are summarized in Table 6. The most commonly reported practices included providing perching and hiding spots at a variety of heights (122/147, 83%), offering multiple places for the cat to hide (118/147, 80.3%) and ensuring adequate space (1–3 m per cat) between food and water bowls, litter boxes, and perching or hiding areas in multi-cat households (112/147, 76.2%).

**Table 6:** Cat owner-reported private space management practices (n = 147)

Provide perching and hiding spots at a variety of heights (ie, furniture, shelves, multilevel cat ‘condo’)	122 (83.0)
Provide multiple places for my cat to hide (ie, covered hiding spots)	118 (80.3)
Adequate space (1–3 m/cat) between food and water bowls, litter boxes and perching/hiding spots in multi-cat household	112 (76.2)
Provide food and water in a quiet space away from noises such as air vents (heating or cooling) and running appliances	83 (56.5)
Provide access to a ‘safe space’ away from other cats with an individualized collar to access food/water or the litter box	27 (18.4)
Decreased the amount of window space my cat could see out of (ie, outdoor animals/cats were bothering him)	9 (6.1)
None of the above describe my cat’s private physical space	2 (1.4)*
I don’t remember/know	1 (0.7)

Data are n (%). Values represent the percentage of cat owners indicating their management practices for each item

* Although ‘none of the above’ was selected to describe their cat’s private physical space, an explanation was not provided in the comment box for these two responses

#### Social interaction management

Detailed cat owner-reported social interaction management practices are summarized in Table 7. The most common practices were provision of massage (or petting) to promote healthy social interaction by 125/145 (86.2%) respondents and play, in general, by 104/145 (71.7%).

**Table 7:** Cat owner-reported social interaction management practices (n = 145)

Massage (or pet) my cat on his terms to promote a healthy social interaction	125 (86.2)
Play a lot with my cat in general	104 (71.7)
Use Feliway (Ceva; feline facial pheromone) spray or diffuser	45 (31.0)
Other cats living in the home can cause stress to my cat, so I make changes to try help (inter-cat aggression)	37 (25.5)
People living in the home can cause stress to my cat, so I make changes to try help	33 (22.8)
Dogs living in the home can cause stress to my cat, so I make changes to try help	18 (12.4)
None of the above describe my cat’s social interaction(s) in the home	4 (2.8)*
I don’t remember/know	1 (0.7)

Data are n (%). Values represent the percentage of cat owners indicating their management practices for each item

* Although ‘none of the above’ was selected to describe their cat’s social interaction(s) in the home, an explanation was not provided in the comment box for these four responses

#### Natural behavior management

Detailed cat owner-reported natural behavior management practices are summarized in Table 8. The most common practices utilized by cat owners were the provision of scratching posts in 129/144 (89.6%) households, toys that look like prey in 113/144 (78.5%) households and catnip in 112/144 (77.8%) households.

**Table 8:** Cat owner-reported natural behavior management practices (n = 144)

Provide scratching posts for my cat(s)	129 (89.6)
Play a lot with my cat using toys that look like prey (eg, mouse, feathers)	113 (78.5)
Use catnip in the household	112 (77.8)
Increase the amount of window space my cat could see out of (eg, watching outdoor activities/wildlife prevent boredom)	89 (61.8)
Offer my cat food and/or treats inside food puzzles or toys that look like prey	59 (41.0)
Provide something for my cat to chew on (eg, cat-safe plants/grass, rawhide chews, dried fish, beef or poultry jerky)	53 (36.8)
Provide music or video of wildlife such as birds for my cat(s) to listen to or watch	36 (25.0)
None of the above describe my cat’s engagement in natural behavior(s)	6 (4.2%)*
I don’t remember/know	0 (0.0)

Data are n (%). Values represent the percentage of cat owners indicating their management practices for each item

* Although ‘none of the above’ was selected to describe their cat’s engagement in natural behavior(s), an explanation was not provided in the comment box for these six responses

## Discussion

This survey’s primary goal was to describe cat owners’ perceptions of the MEMO advice that they received to help prevent another episode of O-FIC. As hypothesized, most cat owners were given diet (94%) and water intake (86.2%) advice. Only 56.9% received advice pertaining to litter box management. A minority were provided with guidance related to private physical space (43.7%), social interaction (25.1%) and natural behavior (26.3%) (Figure 1). Satisfaction scores (for thoroughness of advice) were at similar levels for all aspects of environmental management (Figure 2). Compliance rates were high for all aspects of MEMO advice (range 88.9–97.6%) (Figure 3). Veterinarians were reported as the main providers of advice for essentially all aspects of environmental management, while non-veterinarian informational sources were cited less frequently. Finally, most cat owners reported that they had no challenges with respect to implementing the various aspects of MEMO.

Despite the relatively widespread use of MEMO for acute, chronic and recurrent FIC, only limited research has been conducted. The available literature predominantly describes the favorable efficacy of MEMO implementation for presumably non-obstructive FIC. A study of 46 cats with recurrent FIC had significantly decreased client-generated scores for lower urinary tracts signs, fearfulness, nervousness and signs related to the respiratory tract over a 10-month period after implementing MEMO.^10^ Another study of 20 cats (four female and 16 male) with recurrent struvite-related lower urinary tract signs had higher rates of clinical resolution compared with controls when MEMO was implemented in addition to standardized antibiotic therapy and dietary intervention.^11^ Finally, two case reports also support the incorporation of MEMO into behavioral management of stress and lower urinary signs.^12,13^

With respect to perceptions of advice being provided for environmental management, a single survey capturing the perspective of 606 US veterinarians has been published.^14^ It found that in 75% or more of non-obstructive FIC consultations, veterinarians took history regarding diet (91% of the time), feline stressors (89% of the time), resource setup (77% of the time) and cat–human interaction (31% of the time). It was also observed that the top three veterinarian-reported FIC treatments differed for acute and chronic manifestations: analgesics (89% of the time), modified litter box management (72% of the time) and synthetic feline pheromones (70% of the time) for acute vs prescription diets (86% of the time), modified litter box management (84% of the time) and environmental enhancements (81% of the time) for chronic.^14^ Those authors concluded that cat–human interaction may be under emphasized as a part of the MEMO plan for either presentation.^14^

Our survey is unique in that it explores both the client perspective in terms of MEMO advice and the context of O-FIC. MEMO as a management tool for O-FIC has not been adequately investigated, making this survey one of the first to explore client perceptions of environmental enrichment advice after managing a urethral obstruction. These perspectives are important because, as evidenced by Krause et al,^14^ veterinarians tend to be more likely to recommend more extensive environmental modifications for FIC in its chronic form, and do not as commonly explore cat–human social interaction. Although not yet studied, it is reasonable to assume that a similar approach might be taken when advising in cases of O-FIC. This might have significant repercussions in that O-FIC is both potentially life-threatening and intensely financially and emotionally taxing for cat owners, which would imply that waiting for a case to become recurrent or chronic could be detrimental with respect to outcome. The results of our survey generally fit with those of Krause et al,^14^ in that certain aspects of MEMO are apparently emphasized to cat owners, while others are not recommended as readily. It may be the emergency context in which O-FIC patients present to the veterinary clinic that makes a complete environmental assessment difficult to provide.

The results of this survey strongly support the role veterinarians play in delivering all aspects of advice pertaining to environmental enrichment. It is apparent that clients perceive veterinarians as the experts for information pertaining to all aspects of MEMO advice (51.2–72.0%). Cat owners also complied readily with all aspects of MEMO advice, with compliance rates in the range of 88.9–97.6%. It was hypothesized that more clients would comply with diet, water intake and litter box recommendations as, in the authors’ experience, these are commonly advised by the veterinary community. Alternatively, other aspects of MEMO, such as private physical space, social interaction and natural behavior, may not be as commonly or confidently recommended. The way in which recommendations for at-home environmental enrichment are prioritized by those providing advice may influence the caregivers’ perception. This could make caregivers skeptical of these less commonly discussed aspects of MEMO, especially after the experience of dealing with a urethral obstruction. Instead, clients reported that they readily adopted all aspects of MEMO advice. Most respondents also indicated high levels of satisfaction with the thoroughness of advice provided for all aspects of MEMO. It was anticipated that clients would be more satisfied with advice that veterinarians commonly provided, namely dietary, water intake and litter box advice, compared with aspects of MEMO that are not routinely emphasized, namely those pertaining to private physical space, social interaction and natural behavior. Instead, cat owners reported similar median satisfaction scores in the range of 77–82 for all aspects of MEMO advice (Figure 2).

In terms of challenges encountered while attempting to implement MEMO, most clients reported no challenges (Table 3). The most challenges reported were with dietary recommendations, with 49.6% experiencing difficulties, while 50.4% perceived that there were ‘no challenges’. The top three dietary challenges reported were as follows: cost (29.8%), initial dietary acceptance (18.4%) and impracticality in a multi-cat household (11.3%).

Another interesting finding was that most clients indicated their cats eventually accepted all six aspects of MEMO even if, at first, this was not the case (Table 3). This observation might be explained by a responder bias as many of the cat owners responding to this survey are likely to be a unique group who are actively engaging and seeking information to implement MEMO. Recommendations pertaining to diet (18.4%), water intake (16.5%), social interaction (29.0%) and natural behavior (18.9%) were identified by cat owners to be initially challenging, unlike litter box management (4.1%) and private space (5.4%). Identifying those aspects of MEMO that might be more challenging for initial adoption warrants further investigation to better assist cat owners, as the effort is worth it if the recommendations can be eventually implemented. For example, certain aspects of MEMO had a higher proportion of clients reporting that the main source of the advice was the internet, such as social interaction (24.4%) and natural behavior (26.2%). These might represent two areas in which veterinarians could improve in their MEMO recommendations for O-FIC.

The need for improvement in how MEMO advice is provided is also observed in how the cat owner determines successful implementation. Although many cat owners reported high compliance and minimal to no challenges with MEMO recommendations, this should not be interpreted as no room for improvement. The specifics of how MEMO recommendations are being implemented clearly illustrate this concern. For example, litter box management practices would ideally include the use of more toileting areas than the number of cats, but most owners in this study report having the same number of trays (48.5%) or fewer (11.7%) than the number of cats rather than more (39.8%). Optimized litter box management would also involve multiple litter box locations within the home rather than one, yet slightly less than half of the respondents reported multiple (46.3%) toileting sites and the remainder indicated a single (52.4%) location. Another example involves water intake management, which would preferably utilize more water bowls than the number of cats in the home, but most cat owners reported having the same number of water bowls as cats (42.4%) or fewer (20.0%) rather than more (37.6%).

There are several limitations associated with this survey. A formal definition of natural feline behavior(s) was not established. For part 2 of the survey, only some of the management practices could be explored because of concerns about the length of the survey impacting on response rates. For example, additional dietary questions investigating how the cats were being fed (ie, one big full bowl, hunting/puzzle feeders, microchip-activated individual feeders in multi-cat homes and multiple small meals) and litter box inquiries (ie, size of litter box, type of litter, depth of litter, frequency of cleaning) could have also been included. The number of respondents (n = 167) was relatively small compared with the 606 veterinarians who responded to the non-obstructive FIC survey.^14^ The diagnosis of a urethral obstruction secondary to FIC (vs other causes of obstruction) that was ultimately treated medically cannot be confirmed through medical records. Participant/responder bias undoubtably plays a significant role in our findings: internet/email/social media access, time to answer the questions, favorable opinions of MEMO and engagement, both with respect to modifying the home environment and to providing feedback to the veterinary community. A non-participant/non-responder bias must be considered as the voice of clients who did not participate may not be reflected in the results of this survey, which limits the generalizability to a wider population of cat owners. Self-reporting bias could play a role in both recall and social desirability/peer pressure. It is possible that the type of respondents in this type of survey may want to provide answers that are deemed acceptable, but the anonymous online nature of the study reduces this barrier to providing frank feedback.

## Conclusions

MEMO advice is reaching some caregivers of cats who have been diagnosed with O-FIC. Not all aspects of environmental enrichment are emphasized to cat owners. Diet, water intake and litter box recommendations are provided more commonly than advice related to private physical space, social interaction and natural behavior. Cat owners identified veterinarians as the primary information source for all aspects of MEMO. Compliance rates were reported as very high for all aspects of MEMO, with most caregivers reporting that they did not encounter problems implementing MEMO recommendations. These findings cumulatively highlight the importance of veterinarians as advocates for MEMO.

## Supplemental Material

Supplemental Material Online questionnaire utilized to solicit cat owners’ perspectives.

Table S1 The reasons why cat owners did not comply with MEMO advice.
