# Supplementary material for: Cat owners’ perceptions of multimodal environmental modification advice for obstructive feline idiopathic cystitis
Source: J Feline Med Surg. 2025 Nov 21;27(11):1098612X251381483. doi: 10.1177/1098612X251381483 (PMC12639213; doi:10.1177/1098612X251381483)
Supplement: Supplemental Material [file sj-docx-2-jfm-10.1177_1098612X251381483.docx]

Table 1. Reasons respondents did not comply with various aspects of MEMO advice.

| **Diet** (n=2) |
| --- |
| One Respondent reported:   - that they did not believe diet was playing a role in the cat’s urinary condition - that their cat had another health condition limiting dietary choices   One Respondent:   - did not provide an explanation as to why they did not comply (no response) |
| **Water Intake** (n=5) |
| Three Respondents reported:   - that they were already encouraging water intake   Two Respondents reported:   - that they were already encouraging water intake but didn't believe that water intake was playing a role in their cat’s urinary condition |
| **Litter Box** (n=10) |
| Four Respondents reported:   - that they were already managing the litter box appropriately   Three Respondents reported:   - that they were already managing the litter box appropriately but don't believe litter box management plays a role in their cat’s urinary problem   One Respondent reported:   - that they were already managing the litter box appropriately but don't believe litter box management is playing a role in their cat’s urinary problem - that the litter box recommendations are impractical for cat owners with multiple cats   One Respondent reported:   - that they don't believe litter box management is playing a role in their cat’s urinary problem - that the litter box recommendations are impractical for cat owners with multiple cats - that they had space limitations in their home   One Respondent:   - did not provide an explanation as to why they did not comply (no response) |
| **Private Physical Space** (n=7) |
| Two Respondents reported:   - that they were already providing appropriate private physical space within the home   One Respondent reported:   - that they were already providing appropriate private physical space within the home - that the private physical space recommendations are impractical for cat owners with multiple cats - that their cat does not make use of changes or intended improvements to his physical space within the home   One Respondent reported:   - that they were already providing appropriate private physical space within the home but don't believe that access to individual physical space is playing a role in their cat’s urinary problem - that the private physical space recommendations are impractical for cat owners in terms of time - that their cat does not make use of changes or intended improvements to his physical space within the home   One Respondent reported:   - that they don't believe that access to individual physical space is playing a role in their cat’s urinary problem - that the private physical space recommendations are impractical for cat owners with multiple cats   One Respondent reported:   - that they did not have enough information   One Respondent:   - did not provide an explanation as to why they did not comply (no response) |
| **Social Interaction** (n=3) |
| One Respondent reported:   - that they were already providing appropriate social interactions within the home   One Respondent reported:   - that they don’t believe social interactions are playing a role in contributing to the cat’s urinary problem   One Respondent:   - did not provide an explanation as to why they did not comply (no response) |
| **Natural Behavior** (n=1) |
| One Respondent reported:   - that they did not believe the lack of engagement in natural behavior(s) is playing a role in the cat’s urinary problem - that the natural behavior recommendations are impractical for cat owners with multiple cats |
