# Supplementary material for: Cat owners’ perceptions of multimodal environmental modification advice for obstructive feline idiopathic cystitis
Source: J Feline Med Surg. 2025 Nov 21;27(11):1098612X251381483. doi: 10.1177/1098612X251381483 (PMC12639213; doi:10.1177/1098612X251381483)
Supplement: Table S1 [file sj-pdf-1-jfm-10.1177_1098612X251381483.pdf]

Our Goal

If you have/had a MALE cat that has been diagnosed with a urinary tract obstruction (blockage) resulting in the inability to urinate (cannot pee) that is NOT the result of stricture, stones or cancer, then we want to learn about your experience.

From this 30-to-45-minute online survey, we want to learn about the AT HOME CARE advice you were given. This is also called multimodal environmental modification.

This survey has been approved by the XXX Behavioural Research Ethics Board (Beh-REB #3534). Your participation in this survey is completely voluntary and all of your responses will be kept strictly confidential. Information collected though this survey will be used for research purposes aimed at improving at home care and prevention of urinary blockage.

If you have several MALE cats who have had a urinary tract obstruction, please answer for your most recently diagnosed cat. This survey allows for only one cat per household to be enrolled.

General Information

* 1. What is your cat's name?
[If you have more than one cat that has experienced a urinary blockage please enter the information for the cat that has most recently experienced a urinary blockage.]

2. Which country do you live in currently?

Your Cat's Information

* 3. How old is/was {{ Q1 }}?

- kitten (0 to 6 months)
- junior (7 months to 2 years)
- prime (3 to 6 years)
- mature (7 to 10 years)
- senior (11 to 14 years)
- super senior (15 years or older)
- I don't know

* 4. What breed of cat is {{ Q1 }}?

* 5. Is {{ Q1 }} neutered [desexed/altered/fixed] or Intact?

- {{ Q1 }} does NOT have his testicles [Neutered/Desexed/Altered/Fixed]
- {{ Q1 }} DOES have his testicles [Intact]
- I don't know

* 6. How long have you been trying/did you try to manage the problem medically (without surgery) in an effort to prevent another urinary obstruction?

- less than 2 months
- 2 to 6 months
- 7 to 11 months
- 1 year
- 2 years
- 3 years
- 4 years
- 5 years or more
- I don't remember

* 7. Did you treat your cat medically only or did he also need surgery?

- medically only (no surgery)
- surgery known as a PU (perineal urethrostomy)
- surgery known as a cystotomy (stones removed from the urinary bladder)
- two surgeries: a PU (perineal urethrostomy) and a cystotomy (stones removed from urinary bladder)
- I don't remember/know
- Other (please specify)

* 8. Why do you think your cat developed this problem? [answer can be as short as one word or a longer answer]

Advice You Were Given From Any Source of Information, Whether You Followed It Or Not!

* 9. Were you advised to think about the FOOD you were feeding your cat and consider changing it, as a way to try and prevent another urinary obstruction?
[Things like dry or wet food, urinary or nonurinary food, over the counter or veterinary prescription, etc...]

- ◯ Yes
- ◯ No
- ◯ I don't remember/know

* 10. Were you advised to think about doing things to help your cat DRINK MORE WATER, as a way to try and prevent another urinary obstruction?
[Things like wet food, a water fountain, or the type, location and number of water bowls, flavoured water etc...]

- ◯ Yes
- ◯ No
- ◯ I don't remember/know

* 11. Were you advised to think about doing things to improve your cat's LITTERBOX, as a way to try and prevent another urinary obstruction?
[Things like increased number, new locations, easier access, lower sides so it is easier to get in and out, different type of litter, etc ...]

- ◯ Yes
- ◯ No
- ◯ I don't remember/know

* 12. Were you advised to think about doing things to improve your cat's PRIVATE OR INDIVIDUAL PHYSICAL SPACE, as a way to try and prevent another urinary obstruction?
[Things like hiding places and perches, offering resting places at different heights including low and high up resting places such as (i.e., shelves, cat trees), enough food and water bowls per number of cats, etc ...]

- ◯ Yes
- ◯ No
- ◯ I don't remember/know

* 13. Were you advised to think about doing things to improve your cat's SOCIAL INTERACTION (with people and other animals), as a way to try and prevent another urinary obstruction?
[Things like how to play, interact, pet, and groom your cat, etc ...]

- Yes
- No
- I don't remember/know

* 14. Were you advised to think about doing things to improve your cat's ENGAGEMENT IN NATURAL CAT BEHAVIOR(S), as a way to try and prevent another urinary obstruction?
[Things like mimic hunting with toys that look like prey, food puzzles, scratching posts, toys, etc ...]

- Yes
- No
- I don't remember/know

Main Source of Advice That Guided You The MOST!

Which source of information guided you the MOST for each of these things you can do to improve your cat's home life?
[In other words, which of these sources did you mainly listen to?]

* 15. Which of the following served as the main source of information regarding FOOD decisions for your cat?

* 16. Which of the following served as the main source of information regarding things you could do to help your cat DRINK MORE WATER?

* 17. Which of the following served as the main source of information regarding things you could do to improve your cat's LITTERBOX?

* 18. Which of the following served as the main source of information regarding things you could do to improve your cat's INDIVIDUAL or PRIVATE PHYSICAL SPACE?

* 19. Which of the following served as the main source of information regarding things you could do to improve your cat's SOCIAL INTERACTIONS with other animals and people?

* 20. Which of the following served as the main source of information regarding things you could do to improve your cat's ENGAGEMENT IN NATURAL CAT BEHAVIORS?

SATISFACTION with the Advice

* 21. How satisfied are you with the amount of information and guidance you were given to review your cat's DIET and consider possible changes?

Least satisfaction possible (Least amount of information and guidance possible)

Most satisfaction possible (Most amount of information and guidance possible)

* 22. How satisfied are you with the amount of information and guidance you were given to review your cat's WATER INTAKE (amount of water he is drinking) and consider possible changes?

Least satisfaction possible (Least amount of information and guidance possible)

Most satisfaction possible (Most amount of information and guidance possible)

* 23. How satisfied are you with the amount of information and guidance you were given to review your cat's LITTERBOX(ES) and consider possible changes?

Least satisfaction possible (Least amount of information and guidance possible)

Most satisfaction possible (Most amount of information and guidance possible)

* 24. How satisfied are you with the amount of information and guidance you were given to review your cat's INDIVIDUAL/PRIVATE PHYSICAL SPACE within the home and consider possible changes?

Least satisfaction possible (Least amount of information and guidance possible)

Most satisfaction possible (Most amount of information and guidance possible)

* 25. How satisfied are you with the amount of information and guidance you were given to review your cat's SOCIAL INTERACTION(S) with humans and other pets in the home and consider possible changes?

Least satisfaction possible (Least amount of information and guidance possible)	Most satisfaction possible (Most amount of information and guidance possible)

* 26. How satisfied are you with the amount of information and guidance you were given to review your cat's opportunities to engage in/express NATURAL FELINE BEHAVIORS(S) and consider possible changes?

Least satisfaction possible (Least amount of information and guidance possible)	Most satisfaction possible (Most amount of information and guidance possible)

Which Advice Did You Follow and Then Actually Make the Recommended Changes?

* 27. Did you follow the advice and actually try to make changes to improve your cat's DIET, whether you continued with the dietary recommendation(s) or not? [Things like dry or wet food, urinary or nonurinary food, over the counter or veterinary prescription, etc...]

- ◯ Yes
- ◯ No
- ◯ I don't remember/know

* 28. Did you follow the advice and actually try to make changes to help your cat DRINK MORE WATER, whether you continued to do these things or not? [Things like wet food, a water fountain, or the type, location and number of water bowls, flavoured water etc...]

- ◯ Yes
- ◯ No
- ◯ I don't remember/know

* 29. Did you follow the advice and actually try to make changes to improve your cat's LITTERBOX(ES), whether you continued to do these things or not? [Things like increased number, new locations, easier access, lower sides so it is easier to get in and out, different type of litter, etc ...]

- ◯ Yes
- ◯ No
- ◯ I don't remember/know

* 30. Did you actually follow the advice and try to make changes to improve your cat's PRIVATE OR INDIVIDUAL PHYSICAL SPACE, whether you continued to do these things or not? [Things like hiding places and perches, offering resting places at different heights including low and high up resting places such as (i.e., shelves, cat trees), enough food and water bowls per number of cats, etc ...]

- ◯ Yes
- ◯ No
- ◯ I don't remember/know

* 31. Did you actually follow the advice and try to make changes to improve your cat's SOCIAL INTERACTION (with people and other animals), whether you continued to do these things or not? [Things like how to play, interact, pet, and groom your cat, etc ...]

- ◯ Yes
- ◯ No
- ◯ I don't remember/know

* 32. Did you actually follow the advice and try to make changes to improve your cat's ENGAGEMENT IN NATURAL CAT BEHAVIOR(S), whether you continued to do these things or not? [Things like mimic hunting with toys that look like prey, food puzzles, scratching posts, toys, etc ...]

- ◯ Yes
- ◯ No
- ◯ I don't remember/know

Need a Break for a Couple of Minutes?

Click Next when you are ready to continue!

DIET:
Tell Us About Any Difficulties You Had Making Changes

* 33. Which of the following difficulties have you had while trying to make dietary changes? [Check All That Apply]

- [ ] My cat and I have not had any difficulties with diet change
- [ ] He doesn't eat new foods readily AT FIRST but eventually started eating it
- [ ] He doesn't eat new foods readily so I mix different foods to get him to eat it
- [ ] He doesn't eat new food AT ALL so I could not feed it
- [ ] I had to try many different diets
- [ ] My cat has another health problem that requires he eat another diet
- [ ] Vomiting
- [ ] Diarrhea
- [ ] It is too expensive for those on a limited budget
- [ ] it is too impractical as it requires too much of my time
- [ ] it is too impractical for those feeding multiple cats
- [ ] all of the options are overwhelming
- [ ] I was not given enough information and guidance
- [ ] I do not know/remember
- [ ] Other (please specify)

DIET:
Tell Us About Your Decision NOT To Make Changes

* 34. Why did you decide NOT to make changes aimed at improving your cat's diet? [Check All That Apply]

- [ ] I was already feeding an appropriate diet for urinary health before the urinary blockage happened, so I did not have to change it
- [ ] I do not have enough information to understand and make a decision
- [ ] I don't believe that diet is playing a role in contributing to my cat's urinary problem
- [ ] My cat has another health condition that requires a special diet
- [ ] My cat does not readily eat new foods when I offer them to him
- [ ] My cat has had vomiting or diarrhea when I tried to change the food in the past
- [ ] too impractical as it requires too much of my time
- [ ] too impractical for those with multiple cats
- [ ] too expensive for those on a limited budget
- [ ] too many options to make a good decision - it is overwhelming
- [ ] My cat does not eat new or different foods readily (if at all)
- [ ] I don't know/remember
- [ ] Other (please specify)

WATER INTAKE:
Tell Us About Any Difficulties You Had Trying to Make Changes

* 35. Which of the following difficulties have you had while trying to make changes to your cat's WATER INTAKE?
[Check All That Apply]

- [ ] My cat and I have not had any difficulties with changes to his water intake
- [ ] He doesn't drink readily AT FIRST but eventually accepts the change
- [ ] He doesn't drink AT ALL if I make changes
- [ ] It is too expensive for those on a limited budget
- [ ] it is too impractical as it requires too much of my time
- [ ] it is too impractical for those caring for multiple cats
- [ ] all of the options are overwhelming
- [ ] I was not given enough information and guidance
- [ ] I do not know/remember
- [ ] Other (please specify)

WATER INTAKE:
Tell Us About Your Decision NOT To Make Changes

* 36. Why did you decide NOT to make changes aimed at improving your cat's water intake? [Check All That Apply]

- [ ] I was already encouraging my cat to take in enough water before the urinary blockage happened, so I did not have to make changes
- [ ] I do not have enough information to understand and make a decision
- [ ] I don't believe that water intake is playing a role in contributing to my cat's urinary problem
- [ ] too impractical as it requires too much of my time
- [ ] too impractical for those with multiple cats
- [ ] too expensive for those on a limited budget
- [ ] too many options to make a good decision - it is overwhelming
- [ ] my cat does not drink water readily even with different ways of trying to make him drink more
- [ ] I don't know/remember
- [ ] Other (please specify)

LITTERBOX(ES):
Tell Us About Any Difficulties You Had Trying to Make Changes

* 37. Which of the following difficulties have you had while trying to make changes to the LITTERBOX MANAGEMENT?
[Check All That Apply]

- [ ] My cat and I have not had any difficulties with making changes to the litterbox management
- [ ] He doesn't use the litterbox readily AT FIRST but eventually accepts the change
- [ ] He doesn't use the litterbox AT ALL if I make changes
- [ ] It is too expensive for those on a limited budget
- [ ] it is too impractical as it requires too much of my time
- [ ] it is too impractical for those caring for multiple cats
- [ ] all of the options are overwhelming
- [ ] I was not given enough information and guidance
- [ ] I do not know/remember
- [ ] Other (please specify)

LITTERBOX(ES):
Tell Us About Your Decision NOT To Make Changes

* 38. Why did you decide NOT to make changes aimed at improving the litterbox management? [Check All That Apply]

- [ ] I was already managing the litterbox properly, so I did not have to make changes
- [ ] I do not have enough information to understand and make a decision
- [ ] I don't believe that the litterbox is playing a role in contributing to my cat's urinary problem
- [ ] too impractical as it requires too much of my time
- [ ] too impractical for those with multiple cats
- [ ] too expensive for those on a limited budget
- [ ] too many options to make a good decision - it is overwhelming
- [ ] my cat does not use the litterbox readily even with different ways of trying to encourage him to use it
- [ ] I don't know/remember
- [ ] Other (please specify)

INDIVIDUAL or PRIVATE PHYSICAL SPACE:
Tell Us About Any Difficulties You Had Trying to Make Changes

* 39. Which of the following difficulties have you had while trying to make changes to your cat's individual/private space?
[Check All That Apply]

- [ ] My cat and I have not had any difficulties with changes to his individual/private space
- [ ] He doesn't use the additional individual/private space readily AT FIRST but eventually accepts the change
- [ ] He doesn't use the additional individual/private space AT ALL if I make changes
- [ ] It is too expensive for those on a limited budget
- [ ] it is too impractical as it requires too much of my time
- [ ] it is too impractical for those caring for multiple cats
- [ ] all of the options are overwhelming
- [ ] I was not given enough information and guidance
- [ ] I do not know/remember
- [ ] Other (please specify)

INDIVIDUAL or PRIVATE PHYSICAL SPACE:
Tell Us About Your Decision NOT To Make Changes

* 40. Why did you decide NOT to make changes aimed at improving your cat's individual or private physical space within the home? [Check All That Apply]

- [ ] I was already providing appropriate individual physical space in the home for my cat, so I did not have to make changes
- [ ] I do not have enough information to understand and make a decision
- [ ] I don't believe that individual physical space is playing a role in contributing to my cat's urinary problem
- [ ] too impractical as it requires too much of my time
- [ ] too impractical for those with multiple cats
- [ ] too expensive for those on a limited budget
- [ ] too many options to make a good decision - it is overwhelming
- [ ] my cat does not make use of changes or intended improvements to his individual physical space within the home
- [ ] I don't know/remember
- [ ] Other (please specify)

SOCIAL INTERACTION(S):
Tell Us About Any Difficulties You Had Trying To Make Changes

* 41. Which of the following difficulties have you had while trying to make changes to your cat's social interaction(s) with people and other pets? [Check All That Apply]

- [ ] My cat and I have not had any difficulties with changes to his social interaction(s) with people and other pets
- [ ] He doesn't engage in new ways of social interaction(s) with people and other pets readily AT FIRST but eventually accepts the change
- [ ] He doesn't engage in new ways of social interaction(s) with people and other pets AT ALL if I make changes
- [ ] It is too expensive for those on a limited budget
- [ ] it is too impractical as it requires too much of my time
- [ ] it is too impractical for those caring for multiple cats
- [ ] all of the options are overwhelming
- [ ] I was not given enough information and guidance
- [ ] I do not know/remember
- [ ] Other (please specify)

SOCIAL INTERACTIONS:
Tell Us About Your Decision NOT To Make Changes

* 42. Why did you decide NOT to make changes aimed at improving your cat's social interaction(s) with humans or other pets in the home? [Check All That Apply]

- [ ] I was already providing appropriate social interaction(s) in the home for my cat
- [ ] I do not have enough information to understand and make a decision
- [ ] I don't believe that social interaction(s) are playing a role in contributing to my cat's urinary problem
- [ ] too impractical as it requires too much of my time
- [ ] too impractical for those with multiple cats
- [ ] too expensive for those on a limited budget
- [ ] too many options to make a good decision - it is overwhelming
- [ ] it is hard to get my cat to engage in more social interaction(s) with humans in the home
- [ ] it is hard to get my cat to engage in more social interaction(s) with other pets in the home
- [ ] I don't know/remember
- [ ] Other (please specify)

ENGAGEMENT IN NATURAL CAT BEHAVIOR(S):
Tell Us About Any Difficulties You Had Trying To Make Changes

* 43. Which of the following difficulties have you had while trying to make changes to your cat's opportunities to engage in natural feline behavior(s)? [Check All That Apply]

- [ ] My cat and I have not had any difficulties with changes to his opportunities to engage in normal feline behavior(s)
- [ ] He doesn't accept new ways of engaging in normal feline behavior(s) readily AT FIRST but eventually accepts the change
- [ ] He doesn't accept new ways of engaging in normal feline behavior(s) AT ALL if I make changes
- [ ] It is too expensive for those on a limited budget
- [ ] it is too impractical as it requires too much of my time
- [ ] it is too impractical for those caring for multiple cats
- [ ] all of the options are overwhelming
- [ ] I was not given enough information and guidance
- [ ] I do not know/remember
- [ ] Other (please specify)

ENGAGEMENT IN NATURAL CAT BEHAVIOR(S):
Tell Us About Your Decision NOT To Make Changes

* 44. Why did you decide NOT to make changes aimed at improving your cat's engagement in natural feline behavior(s)? [Check All That Apply]

- [ ] I was already providing appropriate opportunities for my cat to engage in/express natural feline behavior(s) in the home, so I did not make changes
- [ ] I do not have enough information to understand and make a decision
- [ ] I don't believe that lack of engagement in natural feline behavior(s) is playing a role in contributing to my cat's urinary problem
- [ ] too impractical as it requires too much of my time
- [ ] too impractical for those with multiple cats
- [ ] too expensive for those on a limited budget
- [ ] too many options to make a good decision - it is overwhelming
- [ ] my cat does not make use of opportunities to engage in/express natural feline behavior(s) within the home
- [ ] I don't know/remember
- [ ] Other (please specify)

____

Do You Have More Time To Answer More Questions?

* 45. Do you have time to answer more questions?

- ◯ Yes, lets keep going!
- ◯ No, I would like to go to the end of the survey and exit

Tell Us About Your Cat's Diet

46. What kind of food is your cat currently eating?
[Check All That apply]

- [ ] A standard commercial cat food (not veterinary prescription)
- [ ] A therapeutic/veterinary prescription food designed for cats with urinary problems (veterinary prescription)
- [ ] cooked home-made (cook it yourself)
- [ ] raw food (home-made or purchased)
- [ ] other (please specify)

Tell Us More About Your Cat's Diet

47. Is your cat currently eating dry or wet food?

- ◯ dry (kibble)
- ◯ wet (tin cat, semi moist, foil tray, pouch)
- ◯ both dry (kibble) and wet (tin cat, semi moist, foil tray, pouch)
- ◯ I don't know/remember

48. Is your cat currently eating a diet designed to help with lower urinary problems?

- ◯ No, he is eating a Non-Urinary diet
- ◯ Yes, he is eating a Urinary diet
- ◯ He is eating a mixture of both a Non-Urinary and a Urinary Diet
- ◯ I don't remember/know

Tell Us About Your Cat's Water Intake

49. How do you provide water to your cat?

- water bowl(s)
- water fountain(s)
- both water bowl(s) and water fountain (s)
- I don't know/remember

50. Do you let a bathroom or kitchen tap drip to allow your cat to drink?

- Yes
- No
- I don't know/remember

51. Do you use a hydration supplement? [examples are Hydra Care or Oralade]

- Yes
- No
- I don't know/remember

52. Do you flavor the water (i.e. tuna juice, other)?

- Yes
- No
- I don't know/remember

Tell Us About Your Cat's Water Bowl(s)

53. What type of water bowl do you use? [Check All That Apply]

- ☐ glass
- ☐ ceramic
- ☐ stainless steel
- ☐ plastic
- ☐ wood
- ☐ I don't know/remember
- ☐ None of the above

54. Do you keep the water bowl filled to the top (brim) of the bowl or partially filled?

- ○ Yes, I typically keep the water bowl filled to the top of the bowl
- ○ No, I typically keep the water bowl filled partially full
- ○ I do not remember/know

55. Is your water bowl 12 centimeters or larger in diameter to prevent contact with his whiskers against the side of the bowl?

- ○ yes
- ○ no
- ○ I don't know/remember

56. How many water bowls do you have compared to the number of cats that you have?

- ○ more water bowls than number of cats
- ○ same number of water bowls as number of cats
- ○ fewer water bowls than number of cats
- ○ I don't know/remember

57. How many locations around the home do you have access to water bowls?

- ○ multiple locations
- ○ single location
- ○ I don't know/remember

Tell Us About Your Cat's Water Fountain(s)

58. If you use a water fountain(s), then how many water fountain(s) do you have compared to the number of cats that you have?

- I do not use a water fountain(s)
- more water fountains than number of cats
- same number of water fountains as number of cats
- fewer water fountains than number of cats
- I don't know/remember

59. If you use a water fountain(s), then how many locations around the home do you have access to water fountains?

- I do not use a water fountain(s)
- multiple locations
- single location
- I don't know/remember

Tell Us About Your Cat's Litterbox

60. How many litterboxes do you have compared to the number of cats that you have?

- ◯ more litterboxes than number of cats
- ◯ same number of litterboxes as number of cats
- ◯ fewer litterboxes than number of cats
- ◯ I don't use a litterbox(es)
- ◯ I don't know/remember

61. How many locations around the home do you have access to litterboxes?

- ◯ multiple locations
- ◯ single location
- ◯ I don't use a litterbox(es)
- ◯ I don't know/remember

Tell Us About Your Cat's Individual/Private Physical Space

62. Which of the following describe your cat's individual/private physical space at the CURRENT time?
[Check All That Apply]

- ☐ I provide adequate space (1-3 meters per cat) between food and water bowls, litterboxes and perching/hiding spots in my multi-cat household
- ☐ I provide multiple places for my cat to hide (i.e. covered hiding spots)
- ☐ I decreased the amount of window space my cat could see out of (i.e. outdoor animals were bothering him such as outdoor cats)
- ☐ I provide perching or hiding spots at a variety of heights (i.e. furniture, shelves, multi-level cat "condo") to provide my cat with a variety of viewing points
- ☐ I try to offer food and water in a quiet space away from noises such as air vents (heating or cooling) and running appliances
- ☐ I try to provide access to a "safe space" away from other cats with an individualized collar to access food/water or the litterbox
- ☐ I don't remember/know
- ☐ None of the above

Tell Us About Your Cat's Social Interaction(s) With People and Other Pets

63. Which of the following describe your cat's social interaction(s) with people and other pets at the CURRENT time?
[Check All That Apply.]

- ☐ I play alot with my cat in general
- ☐ I realize that other cats living in the home can cause stress to my cat so I make changes to try help (inter-cat aggression)
- ☐ I realize that dogs living in the home can cause stress to my cat so I make changes to try help
- ☐ I realize that people living in the home can cause stress to my cat so I make changes to try help
- ☐ I massage (or pet) my cat on his terms to promote a healthy social interaction
- ☐ I use Feliway (Feline Facial Pheromone) spray or diffuser
- ☐ I don't remember/know
- ☐ None of the above

Tell Us About Your Cat's Engagement In Natural Feline Behavior(s)

64. Which of the following describe your cat's engagement in natural feline behaviors(s) within the home at the CURRENT time?

- [ ] I play a lot with my cat using toys that looked like prey (i.e. mouse, feathers, etc...)
- [ ] I increased the amount of window space by cat could see out of (i.e. watching outdoor activities and wildlife prevent him from being bored)
- [ ] I provide music or video of wildlife such as birds for my cat(s) to listen to or watch
- [ ] I try to offer my cat food and/or treats inside food puzzles, or toys that looked like prey
- [ ] I use catnip in the household
- [ ] I provide something for my cat to chew on (appropriate cat-safe plants or grass, moistened rawhide chews, dried fish, beef or poultry jerky, etc...)
- [ ] I provide scratching posts for my cat(s)
- [ ] I don't remember/know
- [ ] None of the above

THANK YOU

Your willingness to participate in our survey is greatly appreciated!

65. How could the advice you were given on how to care for your cat AT HOME in an attempt to try and prevent another urethral obstruction be IMPROVED upon?
[This question is not required to successfully finish the survey.]
